# [Expression of Concern] DTX3L is upregulated in glioma and is associated with glioma progression

**DOI:** 10.3892/ijmm.2026.5816

**Published:** 2026-03-30

**Authors:** Peng Xu, Xuelei Tao, Chengjin Zhao, Qingfeng Huang, Hao Chang, Na Ban, Yuanqi Bei, Xiaojie Xia, Chaoyan Shen, Kun Wang, Li Xu, Peizhang Wu, Jianbing Ren, Donglin Wang

Int J Mol Med 40: 491-498, 2017; DOI: 10.3892/ijmm.2017.3023

Following the publication of the above paper, it was drawn to the Editor's attention by a concerned reader that, regarding the paraffin-embedded glioma tissue sections shown in Fig. 2A on p. 494, the Ki-67/Grade III and DTX3L/Grade IV data panels contained an overlapping section, suggesting that these data panels were derived from the same original source where different experimental groups were reported. In addition, GAPDH control western blots featured in Figs. 1C and 4E were found to be strikingly similar, even though the experimental conditions reported for these figure parts were different.

The authors have been contacted by the Editorial Office to offer an explanation for these apparent anomalies in the presentation of the data in their paper, and we are awaiting their response. Due to the fact that we have been made aware of potential issues surrounding the scientific integrity of this paper, we are issuing an Expression of Concern to notify readers of these potential problems while the Editorial Office continues to investigate this matter further.

